# Dental Metallurgy

**Published:** 1882-12

**Authors:** 


*Dental Metallurgy.* A manual for the use of Dental Students. By Chas. J. Essig, M. D., D. D. S., Professor ot Mechanical Dentistry and Metallurgy in the Dental Department of the University of Pennsylvania. Publishers: S. S. White Dental Manufacturing Company, Philadelphia, 1882.

This is another valuable contribution to the text books of the profession, and contains information of the greatest importance to the dental student and practitioner. Dr. Essig has compiled a complete and readily comprehended description of the origin, nature properties and alloys of the greater number of the metals employed in dental practice, and given many valuable formulæ. We notice however an omission of descriptions of bismuth and antimony, although the formulæ for fusible metals, type metals, &c, are given.

The use of zinc counter-dies in connection with zinc dies, is highly recommended in the process of swaging plates, and much interesting matter, the result of considerable research is presented in a condensed form, very well adapted to the needs of dental students.

